# Implementing Population Health Management (PHM) Across an Integrated Care System in England, Using Action Learning Sets and an Embedded, Formative Process Evaluation

**DOI:** 10.5334/ijic.10027

**Published:** 2026-04-24

**Authors:** Julian Elston, Felix Gradinger, Tahir Bockarie, Kristian Tomblin, Ginny Snaith, Richard Byng, Sheena Asthana

**Affiliations:** 1 Community and Primary Care Research Centre, University of Plymouth, United Kingdom; 2 Plymouth Health Determinants Research Collaboration, United Kingdom; 3 Honorary Global Health Researcher, King’s College London, United Kingdom; 4 Devon County Council, United Kingdom; 5 NHS Devon Integrated Care Board, United Kingdom; 6 Centre for Health Technology, University of Plymouth, United Kingdom; 7 Centre for Coastal Communities, University of Plymouth, United Kingdom

**Keywords:** population health management, primary care, integrated care, embedded research, linked datasets, health inequalities

## Abstract

**Introduction:**

Population Health Management (PHM) is a UK priority for Integrated Care Systems (ICSs), aiming to deliver proactive, preventative, person-centred care using integrated health and care datasets. However, evidence on implementation in primary care remains limited.

**Methods:**

This comparative case study used embedded researchers, ethnography, interviews, and observations to formatively evaluate a 24-month PHM programme across 31 Primary Care Networks in one ICS. Data were thematically analysed using Excel and NVivo, with findings fed back to participants to guide programme delivery.

**Results:**

Around 200 stakeholders participated in Action Learning Sets, fostering cross-sector collaboration within four localities. Few innovations, developed using integrated datasets, progressed to delivery, limiting their impact on patient health. While PHM infrastructure was established, delivery was constrained by operational pressures, data governance challenges, limited resources, lack of strategic integration and the nature of local relationships.

**Discussion:**

Effective PHM implementation requires more than infrastructure and governance. It depends on developing system-wide soft skills (facilitation, co-production), motivating stakeholders, and investing in processes that support insight generation, innovation piloting, and evidencing of impact.

**Conclusion:**

The study highlights the need for stronger strategic integration, sustained resourcing, coordination, and co-production to realise PHM’s potential at system, place and neighbourhood levels in addressing health inequalities and improving population outcomes.

## Introduction

In the UK, Europe, and USA, rising demand on health and social care systems combined with financial and workforce pressures [1,2] has led to increasing calls for a Population Health Management (PHM) approach to redesigning services in an integrated, whole system way. PHM aims to improve patient experience, population health and reduce inequalities by using linked electronic patient records (EPR) from different providers to design more targeted, preventative, and person-centred services [3,4]. In this sense, PHM brings together two of IFIC’s nine pillars of integrated care; population health and digital solutions.

In England, PHM has been promoted since 2018 [4] and is now embedded in national policies such as Core20PLUS5 health inequalities strategy, Universal Personalised Care, Anticipatory Care and the Community Mental Health Framework. Integrated Care Systems (ICSs) are responsible for implementing PHM at system, place and neighbourhood (Primary Care Network) levels, with growing expectations for primary and out-of-hospital care to play a proactive, preventative role [5,6].

**Figure 1:**
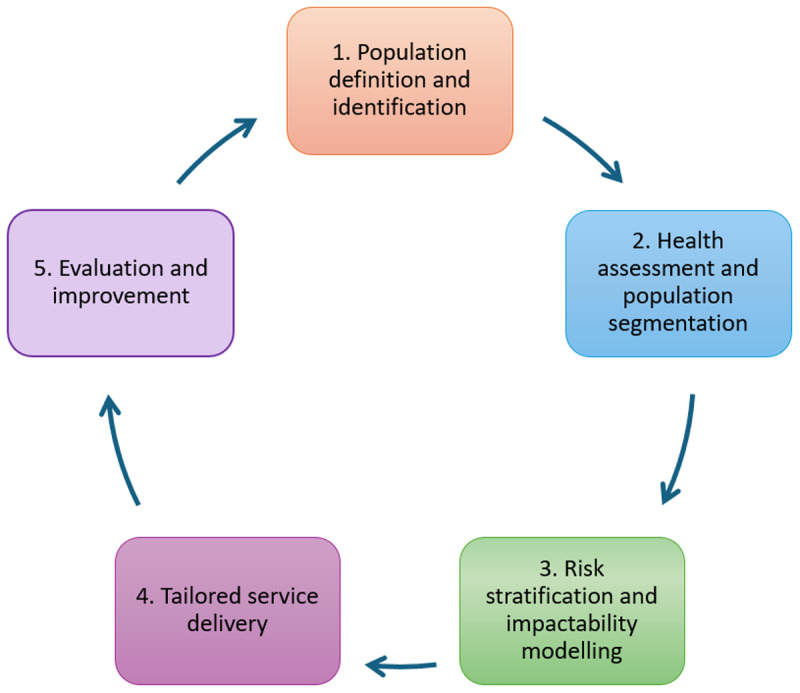
The PHM cycle [5].

PHM involves linking and analysing historic health, social care and wider social determinant data at patient and population levels. Patients or communities can be segmented or stratified to identify those ‘at risk’ of poor outcomes or unmet needs [7,8,9], informing tailored service responses. These can be tested and evaluated for their impact on health, patient experience, inequalities, workforce capacity and costs [10] – the ‘Quintuple Aim’ [4] – completing the PHM cycle (Figure 1) [5,11].

**Figure 2:**
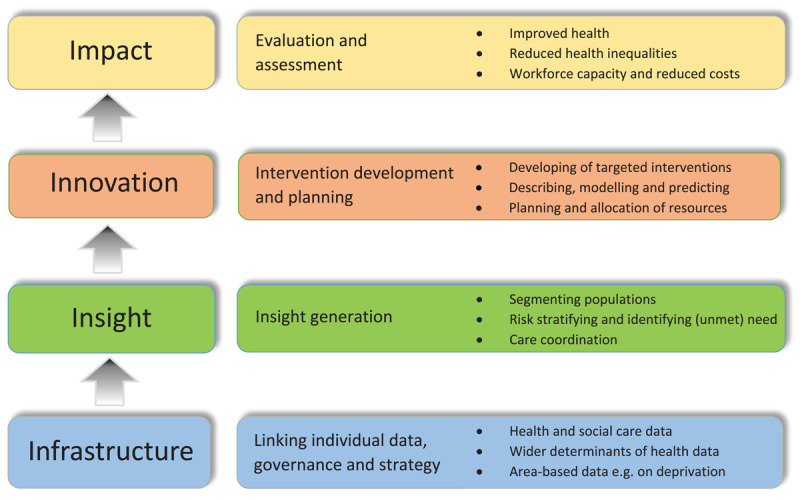
Building blocks for PHM implementation. Adapted from Main et al (2022) and Siegel et al (2019) [13,14].

Such steps are reflected in NHS England’s model of PHM implementation, summarised as: i) the development of an *Integrated* dataset; ii) *Insight* generation; and iii) targeted *Innovation* development. A fourth step, advocated in two recent literature reviews [12,13,14], notes the importance of demonstrating iv) *Impact* on health, inequalities, and the quality and person-centredness of care. Together, these critical building blocks form what might be called the 4 ‘I’s framework. Figure 2 illustrates this process.

Arguably, PHM is another approach to needs assessment in public health, albeit based on individual rather than area level data. Individual NHS data has been underused historically for improvement purposes [10]. With the current interest in ‘Big Data’ [15] and recent policy, structural and legal changes (e.g. Federated Data Platforms, shared electronic records), PHM offers commissioners and health and social care providers a means of basing local policy on more granular data, supporting NHS policy objectives [16].

However, evidence on how to implement PHM is still emerging. At system level, this largely comes from a dozen studies from the USA, Canada, Singapore and Europe [17,18], often in the context of insurance-based health care provision [9,19]. These suggest that implementation, as conceptualised in Figure 2, may be challenging. For example, building integrated infrastructure can present regulatory, technical, methodological and cultural issues [15,19,20]. Segmentation and risk stratification may be hindered by a lack of digital and analytical skills [13]. Cross-national implementation studies, including in Greater Manchester, UK, point to the importance of investing in and developing the ‘softer’ aspects of PHM – building a vision and relationships, working collaboratively, paying attention to governance, transparency and issues of trust [21]. Findings include the importance of an enabling learning environment that facilitates the workforce (managers/clinicians), patients and other stakeholders to co-design innovations and support implementation [17,21]. Insight and innovation development may require skills such as networking, quality improvement, and change management if insights are to be turned into innovations that deliver benefit, and evaluation skills to demonstrate impact [13,14].

As well as being relatively sparse, UK evidence relating to PHM implementation is based largely around meso-level case studies, often relating to single secondary or primary care provider datasets rather than linked datasets and multidisciplinary teams (functional and clinical integration) [22,23]. Yet, evidence suggests that, far from being a simple, rational, linear process to implement, PHM is a complex intervention involving macro, meso and micro contexts and mechanisms [17,18,19,21] i.e. a multi-dimensional programme requiring structural (vertical/horizontal), functional, clinical and service integration [24].

Finally, few studies have sought to understand PHM implementation as happening in a real-world setting, within a complex adaptive system. Subtle differences in local context and circumstance challenge positivism claims of causality. Understanding such variations enables a focus on transferability of findings to other areas rather than generalisability [25,26].

Against this background, this paper presents a pragmatic, participatory, formative process evaluation of an English ICS’s PHM implementation programme across its Primary Care Networks (PCNs). This was commissioned by the ICS in recognition of the need for an adaptive learning approach given the lack of evidence, the complexity of the integration context, with efforts somewhat floundering despite integrated care pioneers in 2015 and the creation of ICSs in 2021 [27] and on-going financial pressures.

The programme was based on a previous national NHS England PHM development programme (provided by a USA-based commercial partner, Optum) to four of its 31 PCNs and one of its four locality care partnerships (LCPs) in the ICS during 2020–21. As an early adopter, acceptance onto this programme required a degree of infrastructure development and data linkage. This study evaluated the ICS’s revised, shortened version of Optum’s PHM programme. It had a similar format and structure, but comprised less Action Learning Sets (ALSs) and was delivered by Health Innovation Southwest (HIN SW) over 20 weeks across all its localities and PCNs between April 2022–24.

The programme aimed to provide formative learning throughout the two-year period, as well as potential summative learning from innovations implemented in this period. As ultimately no innovations were delivered, this paper, therefore, reports on the formative learning, addressing the research question: What factors facilitate or hinder PHM roll-out at PCN level across the ICS?

## Methodology

### Setting and PHM background

The study was based in an English ICS with a population of ~1,250,000 people, characterised by rural areas and several coastal towns and cities, most struggling with deindustrialisation, poverty, ageing populations and growing health inequalities [28]. The ICS has 121 GP practices organised into 31 PCNs (covering ~30–50 k people each) across four localities, renamed A (with 4 PCNs), B (11), C (8), and D (8) to maintain anonymity.

Work on developing the PHM infrastructure i.e. a dataset linked at the individual level, started in 2020, underpinned by a set of information governance principles, revised in 2022 to meet national guidance [29]. This sought to link data from 31 PCNs, four acute hospital trusts, one adult mental health trust, and three community and three social service organisations, to create a non-live dataset on ~900,000 patients, containing ~700 data fields (demographics, clinical tests, diagnosis, treatment and some lifestyle and health outcomes) with historical data spanning 4–11 years, updated quarterly. Access was controlled by a stakeholder Use Governance Board.

### Study design

This comprised a formative process evaluation and comparative case study of the ICS’s four localities, using embedded university researchers (JE, FG, TB) [30] holding honorary ICS contracts. They participated in internal PHM governance committee meetings, the PHM delivery group, all ALSs workshops and related events. Acting as participant observers, they served as ‘critical friends’, providing challenge, feedback and learning throughout the study. The researchers were supervised by two external academics (RB, SA).

This approach is grounded in systems thinking and supports innovation by collecting and analysing data with participants to collectively improve programme design and implementation in near real time [31], not dissimilar to developmental evaluation [32] and participatory action research [33].

### The ICS PHM programme

The aim of the ICS programme was to support PCNs identify a local health or service issue (using the linked dataset), and, in response, develop and pilot an innovation. To do this, the ICS funded six structured two-hour ALSs for PCNs in four localities over a 2-year period, delivered face-to-face and online, using MS Teams. ALSs were supported by three ICS Business Intelligence (BI) staff, four PHM Coordinator posts (one per locality), a communications person and one external ALS facilitator. Delivery was overseen by the PHM delivery group and manager. GP practices were incentivised to participate with £5,000 to backfill staffing costs. Additional funding to cover Voluntary Community and Social Enterprise (VCSE) engagement was negotiated independently in two localities, with an ambition to bring lived experience to collective sense-making and innovation design.

BI staff developed locality and PCN-level Microsoft Power BI™ dashboards stratified by age, sex, mental and physical health, chronic (multiple) conditions, complexity and Index of Multiple Deprivation (2019).

PHM Coordinators encouraged and supported PCNs, statutory, non-statutory and VCSE sector partners to participate.

An HIN SW facilitator guided ALS discussions through the process of developing a logic model, starting by using dashboards to generate insights and stimulate discussion on potential actions. At least one idea was selected by group consensus for development into an intervention, identifying required inputs, activities, and expected outputs and outcomes.

### Primary, qualitative data collection

Process data involved recording meetings and attendee numbers, stakeholder sectors and roles, and the number of linked dataset requests. Sampling was largely opportunistic, aiming to achieve maximum participation and variation across stakeholder groups by purposefully offering consent to anyone delivering or participating in the programme (no formal inclusion/exclusion criteria). Opportunistic qualitative data included semi-structured interviews with stakeholders (via MS Teams) (n = 10); participation in and observation of naturally occurring events (i.e. all ALS sessions and additional workshops (n = 25); PHM and health inequalities governance and team meetings (n = 11); dedicated feedback and reflective team sessions (n = 8); locality Local Care Partnership events (n = 2); national PHM webinars (n = 10); and expert workshops on the use of linked datasets (n = 2), as well as document analysis. Interviews were transcribed by MS Teams, checked for accuracy, and summarised with key points and reflections. Reflective field notes were recorded during and directly after observations. Note-taking and interviews were guided by topic guides.

### Data analysis

**Figure 3:**
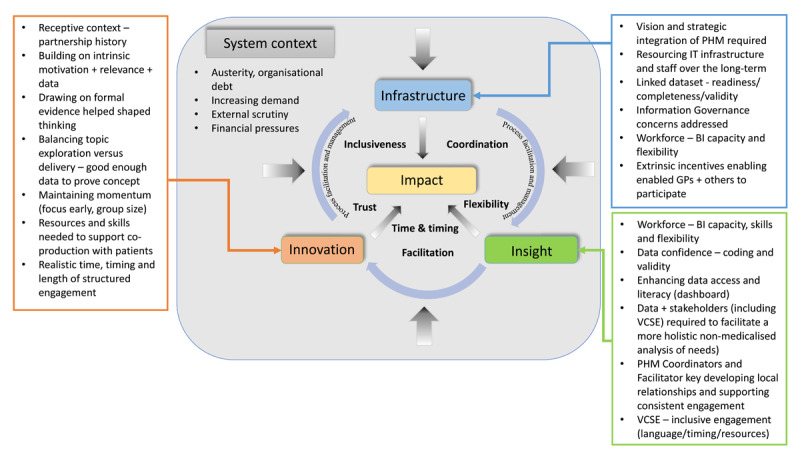
Revised PHM framework showing factors influencing implementation. Key: ALS = Action Learning Sets, BI = Business Intelligence, GP = General Practice, ICS = Integrated Care System, IMD = Index of Multiple Deprivation, LA = Local Authority, PCN = Primary Care Network, PHM = Population Health Management, PPIE = Patient and Public Involvement and Engagement, VCSE = Voluntary, Community and Social Enterprise, WDH = Wider Determinants of Health.

The study undertook a formative analysis of why the programme had or had not achieved its inputs, activities, outputs and outcomes comparing separate implementation, progress and contextual factors across the four localities. Qualitative data was coded in real time inductively and deductively at the study’s interim and final reporting stages (informed by a blended coding framework based on principles identified in a Dutch implementation study [19] and adapted the 4 I’s framework shown above [4,6,13,14]) by one researcher (TB) and grouped into basic themes [34]. Coding categories were checked by the other researchers (JE & FG) and dissonances discussed and resolved. Codes were grouped into themes and refined through discussion and on-going reflection with the PHM delivery group. Analysis was conducted in Excel and NVIVO software. Themes were used to reflect on, refine and adapt the 4 ‘I’s framework to produce a revised conceptual model of implementation [35] (Figure 3).

### Consent

Consent to be interviewed or observed was sought at least 24 hours prior to ALSs, meetings, or events, or directly after if this was not possible. Researchers also regularly reminded participants of the study before each event and followed up non-responders afterwards with one email. This resulted in 110 clinicians, staff and stakeholders participating in the study (Table 1).

**Table 1:** Research participants by sector.


PARTICIPANT SECTOR	NUMBER OF PARTICIPANTS	%

Primary Care Networks	25	23%

VCSE organisations	19	17%

General Practitioners	14	13%

Acute providers^a^	15	14%

NHS commissioners	17	15%

Local authority/Public Health	10	9%

Pharmacy	3	3%

Health Innovation Network^b^	3	3%

Other providers^c^	2	2%

Police	1	1%

Other	1	1%

**Total**	**110**	**100%**


*Note*: ^a^: includes the mental health provider; ^b^: formerly the Academic Health Science Network; ^c^: includes drugs and alcohol and social care providers. Key: VCSE = Voluntary, Community and Social Enterprise.

### Ethics

This research study was funded by a Southwest NHS ICS and received Health Research Authority (HRA) ethical approval on 09.11.2022 (REC reference: 22/SC/0449; IRAS project number: 318324) through ‘proportionate review’ as it excluded patients (they were not involved in the PHM programme). There was no budget for patient involvement in the programme nor the evaluation, which is discussed in the limitations section.

## Results

Table 1 shows just over a third of study participants were from primary care (including GPs), with VCSE, acute providers, public health and NHS commissioners making up most of the remainder.

There were 21 ALSs over 9 months (plus 4 supplementary sessions) with three localities only reaching 5 of the 6 programmed workshops (localities B, C and D). ALS workshops involved 198 attendees (on one or more occasions), varying between 40 to 68 attendees across localities. Overall, two-thirds of attendees (134/198) were not primary care stakeholders. Locality B had the lowest number of attendees and proportion of GPs attending (Table 2). No locality involved patients in ALS workshops, although co-design was being actively considered in locality A.

**Table 2:** Summary of stakeholder attendance at ALSs by locality.


STAKEHOLDER TYPE	LOCALITY				TOTAL

	A	B	C	D	

GP	4	4	5	6	19 (9.6%)

PCN	10	7	20	8	45 (22.7%)

NHS ICS	10	12	18	15	55 (27.8%)

LA (incl. PH)	4	7	7	8	26 (13.1%)

Acute providers	6	5	1	2	14 (7.1%)

Other providers	3	2	11	3	19 (9.6%)

VCSE	6	3	6	5	20 (10.1%)

**Total (%)**	**43 (21.7%)**	**40 (20.2%)**	**68 (34.3%)**	**47 (23.7%)**	**198**


Key: GP = General Practice, ICS = Integrated Care System, LA = Local Authority, PCN = Primary Care Network, VCSE = Voluntary, Community and Social Enterprise.

By the end of the study, six project ideas were identified across PCNs, of which four were worked up from a partially completed logic model into a proposed innovation (localities C and D). One innovation was on the verge of piloting (locality A) (Table 3). Locality B did not reach a point of designing an implementable intervention. Three innovations focused on secondary prevention, two on service efficiency and one on primary prevention. Although no innovations were piloted, the logic model work estimated that the interventions could have reached up to 630 patients and one of the service efficiency innovations ~5% of PCN patients.

**Table 3:** Summary of contextual factors, ALS progress and PCN innovations in development.


LOCALITY	SETTING	COLLABORATION HISTORY	ALS COMPLETED	LOGIC MODEL	PROJECT TOPICS	STAGE OF PHM CYCLE REACHED

A	Rural	Strong	6/6	Partial (lacked inputs and outputs definition)	1. ‘Z’ drug dependency	3–4/5

B	Rural	Weak	5/6	Partial (lacked inputs and outputs definition)	2. Suicide and mental health prevention in rural males	2/5

C	Rural/urban	Moderate	5/6	Developed	3. Did Not Attend individuals at practice	2–3/5

				Developed	4. High Frequency Users attending GP practices and Emergency Departments	2–3/5

D	Rural/urban	Strong	5/6	Partial (lacked inputs definition)	5. Supporting children/families living with a drug/alcohol misuser	3/5

				Partial (lacked inputs definition)	6. Over 50’s individuals experiencing social isolation	3/5


The partial implementation of the PHM programme and limited outcomes were explained by several interacting factors, operating at the macro (system context), meso (organisational) and micro (individual and process) levels. These themes surfaced at all stages of implementation – infrastructure development, insight generation and innovation.

This ultimately affected programme impact, leading to a revision of the 4I’s implementation framework as shown in Figure 3. ‘Softer’ enabling process factors were also identified and incorporated: coordination, flexibility, time, inclusiveness and trust.

### System context

The wider system and organisational environment significantly influenced all stages of PHM implementation. Insufficient centralised funding, historic high levels of organisational debt, and poor performance in high-profile service areas led to stringent external scrutiny (Level 4 of the NHS Oversight Framework) [36]. This resulted in a resource squeeze within the ICS, leading to a PHM programme restructure and loss of key staff. This in turn affected the ICS’s capacity to support data analysis, coordinate workshops and engage stakeholders in ALSs, and ultimately PCNs’ ability to generate insight and develop innovations.

### Infrastructure

A lack of a fully developed PHM infrastructure to build, maintain and use linked datasets was a significant influence on programme implementation. This included the lack of a long-term vision and strategic integration of PHM. This resulted in short-term IT resourcing, an incomplete linked dataset, and several information governance challenges. A central theme was related to low levels of trust. Additional programme infrastructure and resources, notably financial incentives and workforce roles (loss and re-deployment of coordinators and facilitators), also influenced implementation.

#### Lack of PHM vision and strategic integration

The absence of a long-term strategy and operational plan for sustainably resourcing and integrating PHM into the ICS and wider system directly and indirectly affected implementation.

At the time of the ALS rollout, the linked PHM dataset was incomplete, making it difficult for ICS leaders to defend the programme while workforce cuts were underway. Key staff were lost (including the programme manager and two PHM coordinators), while others, such as BI staff, were diverted to other priorities:

*“The BI team currently has a limited number of resources to work on requests…with only two free analysts”* (Interview: 29.02.23)

A short-term, siloed approach followed, with PHM treated as a standalone project rather than integrated into ICS system or area priorities:

*“PHM sat alongside other work and was not integrated into existing work of Local Care Partnerships. That jarred to some degree”* (Interview: 27.10.23 (A/B))

As a result, ALS attendees struggled to connect their projects with system priorities, decision-making processes and resource allocation. Links between neighbourhood, place, and other partnership structures remained weak, with connections emerging sporadically, often through ALS attendees already involved in other strategic agendas, such as work on high-frequency service users or suicide prevention.

Midway through implementation, it became clear the ICS also had a separate health inequalities and prevention workstream. Mounting resource pressures prompted a merger of the two, a pragmatic move given their complementary aims. This unlocked access to additional resources, including GP Health Inequality Fellows, PHM Fellows, and library services.

Ultimately, however, with ICS priorities focused on hospital productivity and financial recovery, PHM’s potential was under-recognised, limiting its impact, sustainability and spread.

#### Datasets – readiness and completeness

The ICS began developing a linked dataset in 2017, but without a long-term strategy or sustainable resourcing, progress slowed. Key infrastructure and governance mechanisms were still in development when the ALSs commenced.

Although early dataset development helped secure a place in NHS England’s Wave 2 PHM pilot, data had initially been commissioned externally and brought in-house for the pilot without a central platform or shared information governance framework:

*“[the ICS] has not fully invested in this approach”* (Interview: 29.02.23)

Consequently, the BI team was still building a comprehensive dataset [37] when the PHM programme began, with much of its time spent cleaning, processing and linking data, limiting its capacity to support ALS delivery:

*“[…] there’s just so much repetitive nature of work, of data being copied everywhere.”* (Interview: 29.02.23)

Technical barriers also arose with GP practices using different IT systems. Regular data uploads required the IT provider’s involvement, which was slow and later demanded additional payment. By the time contracts were finalised, the ALS programme had ended, excluding nearly half of local GP practices from contributing data or participating.

#### Information governance

Governance and data protection concerns, particularly around confidentiality and security, influenced ALS progress and were compounded by trust issues between some PCNs and the ICS. This caused programme delays and diverted BI team capacity into renegotiating contracts and data flows.

Although a system-level governance framework was eventually agreed, 5–10% of GP practices remained reluctant to sign up. Addressing this required additional negotiation and reassurance, supported by the Local Medical Committee and Data Protection Officer:

*“A lot of primary care don’t want to give their data to the ICB because they’re scared of being performance managed or they’re scared of us selling on the data.”* (Interview: 29.02.23)

Operational issues, such as removing ‘opt out’ patients before uploads, caused further delays and drew BI team resources away from supporting ALSs, dashboard development, and data requests. Insight generation was also affected, for example, one locality focusing on children’s mental health was hampered when the Children and Adolescent Mental Health Service dataset wasn’t included. The BI team’s dual role in ALS delivery and managing Use Request Board applications further stretched its capacity.

#### Workforce – skills and capability

The BI team played a central role in ALS delivery, actively contributing, adapting and responding flexibly to support process and participant needs. ALSs provided opportunities to showcase data, build relationships by “putting a face to a name” and gather insights about others’ data priorities:

*“Getting other peoples’ perspectives on what’s important and what to report”* (PHM reflective session: 20.09.23)

The team improved dashboard accessibility, hosted extra data sessions, supported training, managed data requests and helped facilitate meetings. However, financial pressures diverted senior leaders to other priorities, reducing BI team capacity and slowing ALS momentum (PHM reflective session: 20.09.23).

PHM Coordinators (PHMCs) and the independent facilitator were also vital. PHMCs convened, coordinated and encouraged attendance, particularly from overstretched primary care practices, while troubleshooting, organising venues and arranging requested extra sessions (e.g. on data/dashboard interpretation).

When two PHMC posts weren’t replaced, ALS delivery faltered, momentum declined and relationship-building suffered:

*“We need to recognise it takes time to build our data and its quality and our ability to co-design and practice and generate innovation, and to build a network between BI team and rest of the system.”* (PHM reflective session: 20.09.23)

#### Incentives

Financial incentives were crucial for securing GP practice participation. With heavy workloads reported, additional funding helped practices backfill staff and attend meetings but not always. As one member of PHM team acknowledged:

*“GPs need an incentive as they are very stressed at the moment. However, we don’t want to make this [participation in PHM] too transactional but transformational. We need to appeal to their intrinsic motivation not extrinsic motivation”* (PHM team reflective session: 20.09.23)

VCSE stakeholder involvement in two localities was also supported by extra funding, recognising that *“co-design does require resourcing.”* However, motivation was driven more by valuing the process than by financial incentives alone:

*“I have to persuade people that it’s worth their time (and not like before)”* (Interview VCSE: 27.11.23 (B))

Yet, concerns about long-term funding for the sector and the lack of strategic integration of PHM remained evident:

*“A VCSE organisation working on mental health is just going under in [market town] but at the same time the [ICS] and others are saying mental health is a priority and we should set up this new project.”* (Interview VCSE: 27.11.23 (B))

Once again, the absence of a joined-up, strategic, and properly resourced approach was clear.

### Insight

ALSs aimed to generate insights by combining PCN-level data analysis, primarily provided through interactive dashboards, with input from clinical and non-clinical stakeholders in a structured process. These insights were intended to inform the development of local innovations. However, several challenges emerged, particularly relating to analytical capacity, data confidence and quality, limited analytical skills, and varying levels of data literacy among participants.

#### Workforce analytical capacity, skills and flexibility

The dashboard developed by the BI team was useful for emphasising the principles of PHM, highlighting health inequalities and enabling ALS attendees to see what data was available beyond practice-level datasets. Dashboards primarily provided descriptive and comparative analysis, with data presented as numbers, percentages and prevalence rates, colour-coded to indicate a PCN’s relative position to others. However, incidence rates, time-trend analyses, and formal statistical comparisons were unavailable (ALS debrief: 04.04.23), limiting the analytical depth.

#### Data confidence – coding and validity

Clinical attendees frequently questioned the dashboards, raising concerns about the validity, reliability, timing and substitutability of data fields and coding across organisations (Notes ALS: 12.10.23 (B)). For example, variations in coding for depression between primary care and mental health services and the use of parasuicide as a proxy for suicide risk, were challenged. Some data also reportedly differed from that held in individual GP practices, leading to scepticism about its accuracy and utility.

For the BI team, this posed a challenge, as it felt obliged to respond to participants’ queries and “big shopping lists” to maintain engagement. However, this risked distracting from the broader programme aims. As one team member asked:

*“What is the end goal?”* (Team meeting: 11.04.23)

Others warned of the risk of getting “bogged down” in detail, with too much emphasis on data potentially diverting attention away from more person-centred and wider determinants of health perspectives brought by stakeholders:

*“GPs are prone to bring their own take on things, often clinical and medically focused, but primary care are not linked in and end up building stand-alone programmes.”* (Interview: 27.10.23 (A/B))

#### Enhancing data access and numeracy skills

Despite the BI team’s efforts to make the dashboard accessible and easy to interpret, many non-NHS ALS participants struggled with access (due to IG restrictions) or interpreting the data, even with a summary view.

Access was especially problematic for the VCSE sector, which required setting up NHS.net email accounts, a time-consuming process, only to face compatibility issues with software and firewalls. While IT and governance issues were addressed, less interactive electronic copies had to be shared:

*“It took a lot of time to sort out”* (Interview: 23.10.23)

Even with these measures, many participants continued to find the dashboards difficult to understand and interpret (PHM reflective session: 20.09.23). Time constraints due to work pressures further limited opportunities for data scrutiny (ALS debrief: 04.04.23; PHM reflective session: 20.09.23). Attendees from across sectors, primary care, statutory, non-statutory and VCSE, appeared to struggle with data interpretation, suggesting low baseline levels of numeracy.

Taking a flexible approach, the BI team organised additional workshops between ALS sessions to address these issues.

#### Dataset content and scope limited holistic needs assessment

As the dashboards were built predominantly on linked service activity data, this shaped the nature of discussions:

*“Using health and social care service data means discussion can often be clinical more than person-focused.”* (Interview VCSE: 30.11.23 (B/C))

The non-inclusion of key datasets reduced the visibility (PHM meeting: 25.04.23) of certain cohorts and issues, limiting exploration of topics such as children’s autism assessments or familial alcohol-related harm. It also raised concerns about the reliability of proxy indicators (ALS3 Debrief: 23.05.23 (D)), for example, using the ‘living alone’ code when discussing social isolation (ALS3: 29.03.23 (A); 03.07.23 (C)).

To develop a more complete understanding of local need, most ALS groups proposed incorporating additional datasets, typically income and housing data (ALS4:14.09.23 (C); 31.08.23 (B)). The involvement of non-NHS stakeholders was also valuable in this regard, bringing broader, person-centred perspectives:

*“There was data explaining by a data person, but the worlds people come from are very different; it doesn’t mean anything to me; I work with people; so it’s a big gap for me… you need to help me see why it matters and where the people are in this.”* (Interview VCSE: 27.11.23 (B))

There was widespread recognition that quantitative data alone would never be sufficient for understanding patients’ holistic needs. Understanding these required complementary qualitative, experiential and contextual data.

### Innovation

Progress from insight to intervention proved more challenging than expected, even where topics were chosen quickly (Localities A & D). Progress was fastest where individuals held strong pre-existing relationships, were intrinsically motivated, took ownership and had access to resources. Innovation development was also supported by the availability of relevant academic evidence (for example building on extensive, previous work on polypharmacy in locality A).

#### Receptive context

In localities with stronger historical partnerships (A & D), priorities were identified quickly, often driven by dominant stakeholders rather than dashboard data (PHM reflective session: 20.09.23). As several GPs put it: “We know our priorities.” For example, in locality A, a deprescribing project for Z-drugs built on local work led by a medicines optimisation director with academic ties, who had previously conducted a survey of prescribers and patients (ALS3: 17.05.2 (A)).

Elsewhere (B & C), tensions emerged between those wanting more data analysis and those ready to decide (PHM reflective session: 20.09.23), requiring careful facilitation. Wider questions were raised about PHM’s purpose: whether it was primarily to showcase linked datasets or to build local partnerships that integrate lived experience and tacit knowledge to understand local needs.

*“Evidence doesn’t just come from the data. It also comes from published and unpublished research and, importantly, from local people and service users.”* (PHM Webinar: 04.05.23)

#### Building on intrinsic motivation

While financial incentives (extrinsic motivation) were important for engaging GP practices in ALSs (less so for VCSE and other partners), sustaining interest also relied on intrinsic motivation. This was supported by allowing PCNs to focus on issues that mattered to them, rather than those issues suggested by BI analysts or innovations that could reduce costs, as the latter reportedly disengaged GPs (ALS2: 02.05.23 (B)).

Intrinsic motivation was most evident in locality A, which made the most progress towards delivery, but was also observed in other localities (B), where one GP joined another locality’s ALSs due to their interest in the chosen topic.

#### Aligning interests and building relations – boundaries and group size

The deliberative process was complicated by the number of PCNs and variation in local contexts, with data priorities not always relevant to all. Managing this diversity was challenging:

*“More than five PCNs in a group feels too many; they need to be more incremental and bespoke and smaller scale.”*

In locality C, the facilitator addressed this by splitting the group. Although ALS attendance was generally good, fluctuating participation required repeated introductions and re-explanations of data.

Building relationships and helping stakeholders feel that they contributed to insights was crucial (PHM meeting: 13.10.22 (C/D)):

*“Actually, it doesn’t matter what we do but how we work collectively building the new future; commonality of approach.”* (Team meeting: 03.05.23 (B/C))

Trust among stakeholders was vital, but varied across localities depending amongst other things on the strength of existing partnership relations locally:

*“Trust is crucial for the magic to work; knowing and acknowledging that trust is important and how difficult it is to create is important for the partners.”* (Interview VCSE: 27.11.23 (B))

A further challenge observed in several localities (A & C) was differentiating between describing a cohort at risk (to develop interventions) and predicting at-risk individuals for intervention targeting (requiring modelling).

Thus, tension existed between topic selection, involving interested stakeholders, and facilitating a process that enabled meaningful contributions to insights, through fostering relationships and building trust. This required skilled facilitation and time (PHM meeting: 13.10.22 (C/D)).

#### Using formal evidence

Academic evidence, sourced by PHMCs, helped shape ideas in two localities (A & C), alongside local data, clinical and stakeholder input and tacit knowledge. In locality C, an academic paper guided the group towards organisational and system-level solutions. In locality A, academic evidence and survey data enhanced insight development. However, limited PHMC capacity curtailed this potential benefit in other localities.

#### Resources and skills to support co-production with patient

All localities recognised the importance of including community, VCSE and patient voices in the ALS process, as these provide *“insights and perspectives that more formal data won’t show.”* Patient involvement was also noted to help ensure appropriate language when discussing sensitive topics, such as deprescribing or medication reviews (ALS6: 16.08.23 (A)). However, no patients or public members were directly involved in insight or innovation development stages in any locality. Instead, VCSE representatives or social prescribers often acted as proxy voices for ‘lived experience’ (ALS3 debrief: 23.05.23 (B); ALS4: 31.08.23 (B)).

For localities that acknowledged this gap (A), the primary barriers to meaningful patient engagement were resource constraints, lack of skills and insufficient time. These requirements were not factored into programme design.

### Impact

As a result of the different combination of factors above, none of the six innovations under development reached implementation. In the absence of ICB resources to support prototyping, gaining PCN ownership was seen as critical to maintaining momentum and achieving impact (Interview: 20.03.24).

*“Ownership is critical and not really cemented yet.”* (PHM reflective session: 20.09.23)

Among the two localities that made the most progress, only locality D piloted its innovation (through a VCSE partner) after the ALS programme ended. This followed splitting elements of the innovation into more manageable parts and support from a small number of committed dual ICB/PCN-funded stakeholders.

## Discussion

This detailed process evaluation of PHM implementation within one English ICS highlights the complex interplay of contextual, organisational and individual factors shaping the translation of a national policy goal into practical reality. These factors interacted in a non-linear fashion across the stages of PHM implementation – Infrastructure, Insight, and Innovation – across system, place, and primary care levels [38]. Each stage was also sensitive to how the PHM programme was managed, facilitated and resourced in terms of coordination, inclusiveness, flexibility, timing and trust. Our locality comparison also showed local context matters, justifying a bespoke and pragmatic approach taken by the PHM programme, the ALS facilitators and the evaluation.

Regarding infrastructure, our findings echo those of previous UK-centric reviews [13,14] and the wider international literature [17,18,19,21], which emphasise that successful PHM implementation requires leaders to articulate a strong, strategic vision for PHM, supported by long-term investment in developing, maintaining and utilising linked datasets effectively [20]. Robust and transparent governance structures around linked datasets and data protection are essential to avoid continual renegotiations that stall implementation. Relational work is needed to build trust with primary care providers and other stakeholders to address concerns about data safety and confidentiality. These are critical issues in this and other sectors, especially insurance-based systems sensitive to financial confidentiality [39], and are vital to ensure comprehensive dataset coverage. Given the significant capacity impact on BI staff who had to spend time renegotiating and reorganising contracts and provider data flows, a clearer national strategy on data governance would be valuable.

Extrinsic motivation in the form of funding proved an effective tool for supporting engagement, particularly for PCNs facing capacity constraints. Our comparative analysis showed that progression to insight generation was influenced by existing collaborative capital, the quality of relationships, local priorities and the intrinsic motivation of stakeholders. Thus, investment in local partnerships and better (structural) integration of local interests with ICS priorities could strengthen implementation by aligning resources and facilitating spread. This needs to be accompanied by ‘soft’ management skills and a flexible process that supports ongoing learning as these were important to managing different interests across PCNs, maintaining stakeholder engagement and creating momentum towards innovation development. This is consistent with factors and principles identified as key to successful implementation elsewhere [17,18,19].

Most PCNs will be using integrated datasets developed by ICSs, as few will have capability or capacity to link practice data themselves. As we have found this currently generally covers health and social care data. Producing more nuanced and holistic insights into patient needs, a key rationale for PHM [40], will require engaging stakeholders from non-statutory and VCSE sectors as well as patients themselves. Effectively leveraging their knowledge and experience to develop integrated clinical pathways requires dedicated resources and development of co-production skills among facilitators, coordinators, and stakeholders.

The importance of externally resourcing pathway co-design, supporting dashboard access and interpretation, providing primary care workforce training and facilitating patient and public involvement must not be understated. This has been noted in other UK PHM programmes, for example, coronary vascular disease and mental illness risk stratification tools developed for some London PCNs and an acute mental health trust [23]. Although innovations in our study did not reach these stages, the London experience showed that a lack of dedicated implementation support and financing were significant barriers to delivering tailored service pathways. Demonstrating impact on patient health outcomes is also challenging [23]. New PHM care pathways will require thorough assessment to ensure they meet PHM goals (and do not inadvertently widen inequalities), providing necessary evidence for business cases supporting wider adoption [20,21].

These findings suggest the need for a more dynamic model of PHM implementation than NHS England’s linear ‘4I’s’ framework [12,13,14]. Based on a learning approach, van Ede et al.’s (2023) implementation model incorporates not only integrated infrastructure and data analysis but also a domain for community and workforce co-design, alongside an emergent implementation strategy grounded in learning cycles [9,17]. Understanding how to align and integrate infrastructure and technology with effective processes and stakeholder engagement will be critical to successful implementation [38]. In this context, PHM should be recognised as multi-level, multi-dimensional integration programme, requiring cultural change and distributive leadership to foster local ownership and action [17]. Leaders must provide vision and adaptive strategy, backed by resources targeted at multiple system levels, to transform organisational and individual behaviours, that is also sensitive to local circumstances [17,18]. Providing ‘social proof’ of PHM successes, for example, through case studies and building a learning community across organisational, clinical, and community levels, based on advocacy champions and continuous improvement, will further support effective spread [18].

### Study strengths and weaknesses

Evaluating complex implementation programmes such as PHM presents significant practical and methodological challenges [41], given their multiple interacting components that require adaptation and flexible delivery. This study contributes to the relatively limited literature on PHM implementation, especially within primary care, by adopting both ‘top-down’ and ‘bottom-up’ perspectives to understand implementation and integration processes [18].

As Researchers-in-Residence embedded within the intervention delivery, we gained rich insights into the dynamic interactions between context and programme throughout implementation [31]. Employing a systems perspective alongside a flexible approach to data collection enabled us to accommodate delays, understand implementation challenges and monitor programme modifications [32]. Nonetheless, our ‘insider’ role as participant observers risked compromising our objectivity. Regular reflective sessions with the programme team and challenge from our university-based supervisors supported our objectivity and critical judgement [31].

However, limited resources, workforce pressures and time constraints reduced opportunities for additional data collection after ALS sessions, particularly with GPs and PCNs, making us more reliant on observations and informal (consented) conversations. As the study focused on one English ICS (albeit encompassing diverse localities and PCN structures), this may limit its transferability.

## Conclusions

Implementing PHM is a national priority for ICSs in England, supporting policies aimed at delivering more preventative, person-centred care and addressing health inequalities. Despite incentives and resources and using a flexible approach to engaging PCNs and stakeholders, raising awareness and analysing locally linked data, the programme evaluated in this paper did not achieve its aims – no locality developed and implemented an innovation within a 2-year period. System pressures, weak integration with ICS priorities, and reduced capacity affected engagement, contributed to delays and led to a loss of momentum.

Given the intention of better harnessing shared data and predictive analytics to support the shift from sickness to prevention, the lessons of this study have wider relevance to the NHS 10-Year Health Plan. Without addressing system integration, IT infrastructure and IG challenges as well as the ‘human factors’ that affect implementation, the potential to create an NHS that is fit for the future may not be realised.
